# The efficacy of concentrated growth factor and platelet-rich fibrin as scaffolds in regenerative endodontic treatment applied to immature permanent teeth: a retrospective study

**DOI:** 10.1186/s12903-023-03164-y

**Published:** 2023-07-14

**Authors:** Jiahua Li, Leilei Zheng, Baraa Daraqel, Jing Liu, Yun Hu

**Affiliations:** 1 Stomatological Hospital of Chongqing Medical University, No.426 Songshibei Road, Yubei District, Chongqing, 401147 China; 2 Chongqing Key Laboratory of Oral Diseases and Biomedical Sciences, Chongqing, China; 3 Chongqing Municipal Key Laboratory of Oral Biomedical Engineering of Higher Education, Chongqing, China

**Keywords:** Concentrated growth factor, Immature permanent tooth, Platelet-rich fibrin, Regenerative endodontic treatment

## Abstract

**Background:**

The aim of this retrospective study was to compare the efficacy of concentrated growth factor (CGF) and platelet-rich fibrin (PRF) as scaffolds in regenerative endodontic therapy (RET).

**Methods:**

Necrotic immature permanent teeth treated with regenerative endodontic therapy during January 2018 to August 2022 were divided into the CGF and PRF groups according to the scaffold. The CGF and PRF groups included 7 and 6 teeth, respectively. The efficacy of regenerative endodontic therapy was analyzed based on the clinical and radiological outcomes at three different follow up periods: T1 (3–6 months), T2 (6–12 months) and T3 (12–24 months). Statistical analysis was performed using the independent T test, Mann-Whitney test and Fisher’s exact test at a significance level of 0.05.

**Results:**

The success rate of each stage in both groups was 100%. Through quantitative comparison of radiographic outcomes, there was no statistically significant difference between the two groups in terms of root development and periapical lesion healing at each stage, except that the increase rate of radiographic root area in PRF group in the T3 stage was above one in CGF group with statistically significance.

**Conclusions:**

Both CGF and PRF had a similar clinical performance regarding resolution of clinical signs and symptoms, periapical lesion healing, and continued root development as scaffolds in RET. Further prospective studies with large samples for longer follow-up periods are needed.

## Background

Regenerative endodontic therapy (RET) is a treatment method based on the concept of tissue engineering, which aim to promote root development of necrotic immature permanent teeth and the regeneration of the pulp-dentin complex [1]. Such three elements of tissue engineering as seed cells, scaffold materials and growth factors have an essential impact on the efficacy of RET [2, 3]. A suitable scaffold can provide a suitable location for seed cells and biological conditions conducive to cell metabolism, and regulate their differentiation and proliferation.

The main scaffolds for regenerative endodontic therapy are autologous blood clot, autologous platelet concentrate, biomaterial scaffold, and so on [4]. At present, blood clots and autologous platelet concentrate are commonly used in clinic [5]. The traditional method in RET is to stimulate apical bleeding, fill the root canal with blood, and use blood clots (BC) as scaffolds for pulp revascularization. Blood clot as a scaffold in RET had a good practicability and success rate [6]. However, it was found that clinically there were quite a few cases with no or insufficient blood clots which were disable to fill the root canal or support the pressure from the crown sealing material, leading to treatment failure [7]. At the same time, for necrotic mandibular premolars caused by developmental dental anomalies, there was a risk of nerve injury by using files to stimulate bleeding outside the apical foramen because of the physiological and anatomical position of mental foramina and root apices [8]. Therefore, it is necessary to find scaffolds to replace blood clots. Endogenous materials replacing blood clots as scaffolds include platelet‑rich plasma (PRP), platelet-rich fibrin (PRF), and concentrated growth factor (CGF). The phenomenon of root development was observed by applying PRF to the RET of necrotic immature permanent teeth, which proved the feasibility of using PRF instead of traditional blood clot [9].

Saccol et al. obtained the third generation of platelet concentrate, concentrated growth factor, by precise variable speed centrifugation with a specific centrifuge [10]. It is a fibrin matrix with high concentration of growth factors and 3D network structure, which promote soft and hard tissue repair and regeneration, by improvement of cell migration and facilitation of neovascularization [11]. CGF may play an role in vascular maintenance and angiogenesis due to its inclusion of CD34-positive cells [12]. Besides CGF with a stronger regeneration ability contains more growth factors and fibrin matrix than PRF on account of the preparation of CGF adopts differential centrifugation with higher separation efficiency [13–15].

Platelet concentrate products have good biological characteristics and are easy to produce. In recent years, they have been widely used in regenerative treatment of oral medicine, such as periodontal tissue regeneration and orthodontic tooth movement [16, 17]. CGF has shown superior potential for tissue regeneration in clinical and biotechnology applications. However, there are still relatively few reports on the application of CGF in RET. The objective of this study was to evaluate and compare the efficacy of CGF and PRF for RET in necrotic immature permanent teeth, and to select an ideal scaffold.

## Methods

### Inclusion and exclusion criteria

The study design and clinical procedures were conducted in accordance with the Helsinki Declaration and were approved by the ethical committee of the Stomatological Hospital of Chongqing Medical University (ID: CQHS-REC-2022(LSNO.106)). Informed consent to participate in the study was obtained from participant’s legal guardian. Through the inquiry of the CGF/PRF centrifuge operating records during January 2018 to August 2022, the cases of patients treated with RET were preliminarily identified.

**Fig. 1:**
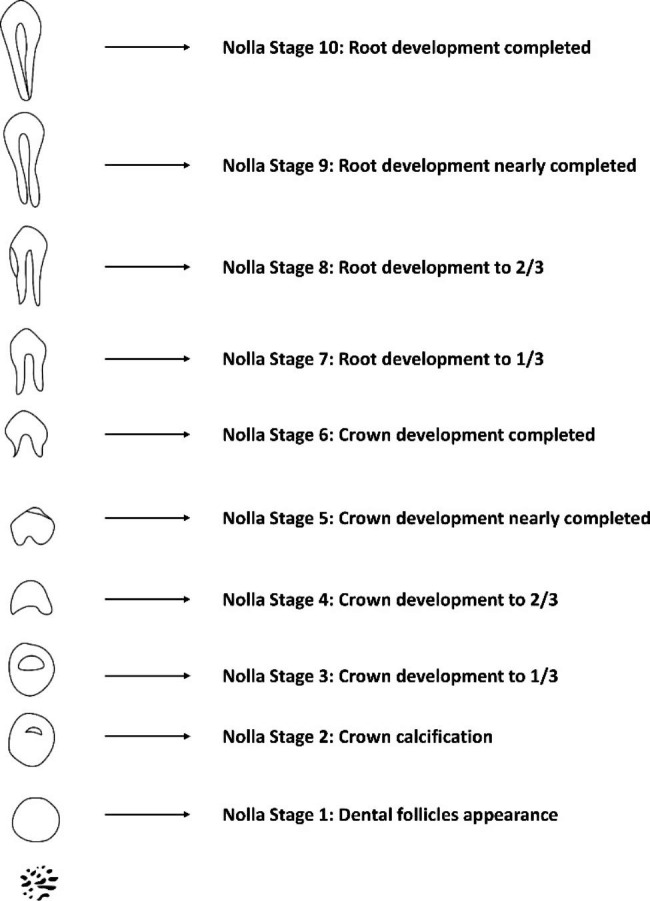
Nolla stage diagram

Clinical records of potential cases were reviewed to determine eligibility for the study with pre-determined inclusion and exclusion criteria. Inclusion criteria: (1) patients had already been treated must be at least 6–16 years old at the time of treatment and the necrotic immature permanent teeth development should be at Nolla 7–9 stages [18]. The Nolla stage was determined by two authors based on Fig. 1 and periapical radiographs. Any disagreement was resolved by discussion with a third author; (2) use CGF or PRF as scaffolds; (3) have complete regenerative endodontic procedures including the final restoration; (4) recall was recorded in clinical records for at least 3 months. Exclusion criteria: (1) serious loss of case data; (2) X-ray image distortion cannot be corrected; (3) According the periapical index (PAI) scoring system [19], teeth with periapical lesion whose PAI scores were not 3–4.

### Data extraction

**Fig. 2:**
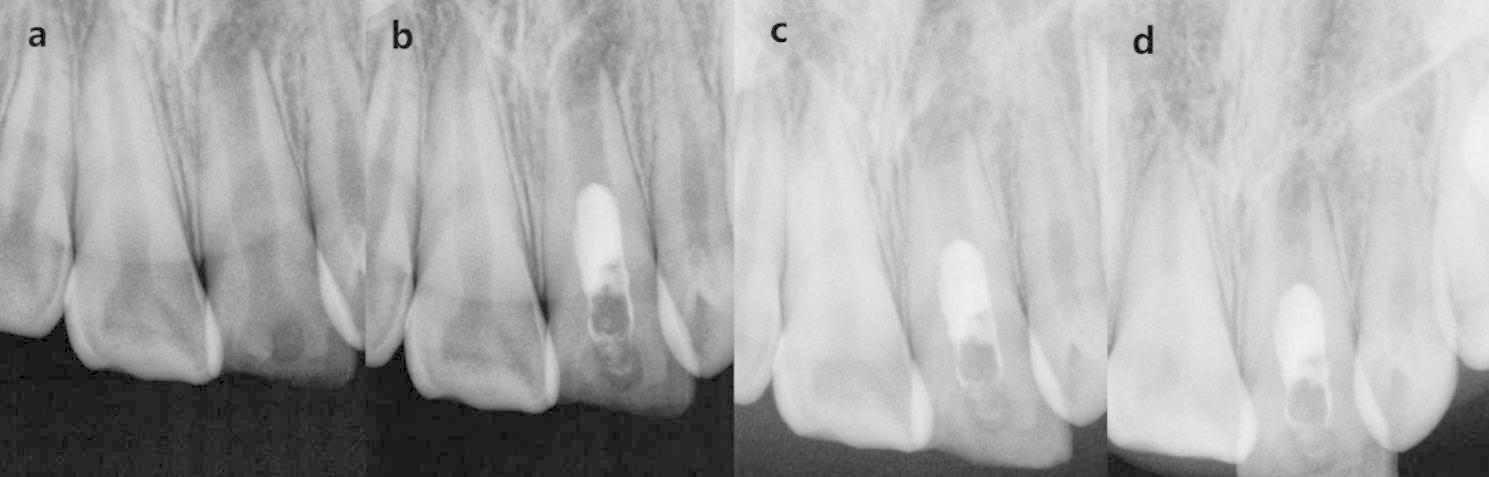
#21 in a 11-year-old boy of CGF group **(a)** The pre-operative periapical radiograph. **(b)** The immediate post-operative periapical radiograph. **(c)** The post-operative periapical radiograph at the 4-month follow-up. **(c)** The post-operative periapical radiograph at the 16-month follow-up

**Fig. 3:**
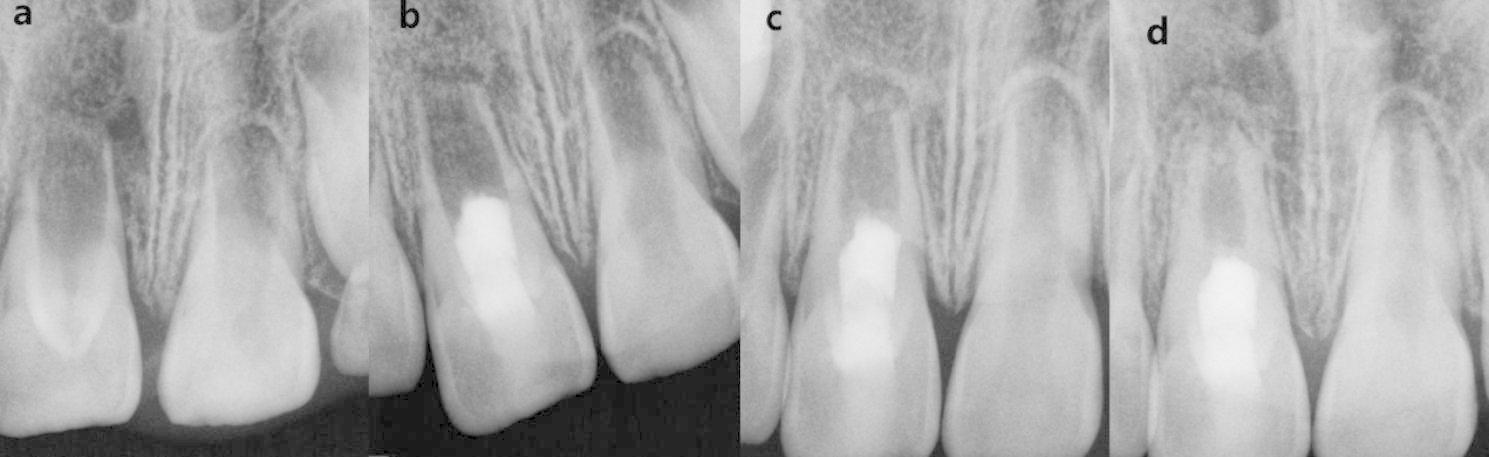
#11 in a 7-year-old girl of PRF group **(a)** The pre-operative periapical radiograph. **(b)** The post-operative periapical radiograph at the 3-month follow-up. **(c)** The post-operative periapical radiograph at the 6-month follow-up. **(c)** The post-operative periapical radiograph at the 15-month follow-up

The included cases were divided into CGF group and PRF group according to the different scaffolds used. Data were extracted from the clinical records of these included cases such as demographic factors, tooth position, etiology, preoperative signs and symptoms, intraoperative treatment details, and follow-up time. Data were recorded and organized using Microsoft Excel (Microsoft Corp., Redmond, W A, USA). The preoperative and follow-up radiographs were taken with the dental X-ray machine (65 kV, 7 mA, and 0.1–0.2 s scan time) using the standardized paralleling technique. All radiographs were saved in jpg format (Figs. 2a–d and 3a–d).

### Preparation of CGF/PRF

**Fig. 4:**
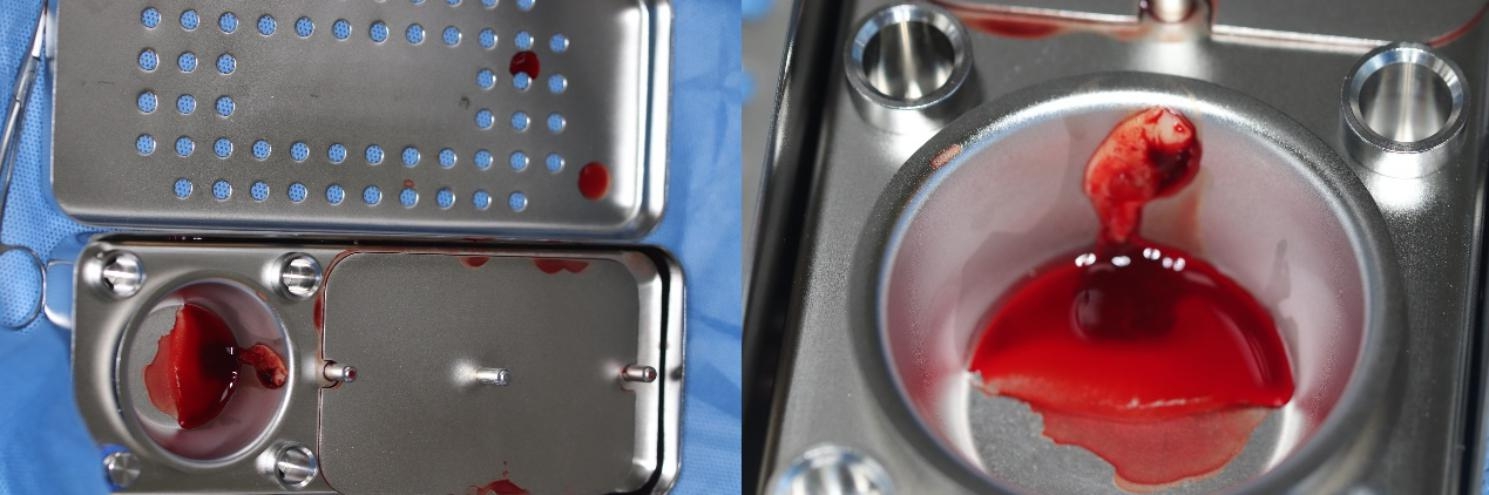
Box-shaped separator, sterile scissor, and CGF clot

Intravenous blood sample (10ml) from the patient was obtained and drawn into a disposable 10 ml nonanticoagulant tube. The tube was placed in a matching centrifuge device (DT-F4, Chengdu Dengtuo Medical Instrument Co., LTD). Depending on the scaffold to be prepared, different centrifugation programs on panel were selected. The PRF was centrifuged at 3000 rpm for 10 min at room temperature. CGF was centrifuged at variable rpm for 15 min, which mainly consist of 2600 rpm for 2 min, 2300 rpm for 4 min, 2600 rpm for 4 min, and 2850 rpm for 3 min. After centrifugation, three distinct layers occurred in the tube: serum layer at the uppermost, CGF/PRF layer at the middle, and red blood cell layer at the bottom. The fibrin gel from the middle layer was separated and put into a specially-made and box-shaped separator to make into a membrane (Fig. 4). The CGF/PRF membrane was then trimmed to a suitable size to match the volume of root canal.

### Treatment procedure

Although this was a retrospective study spanning several years and no specific clinical protocol was implemented before the implementation of treatment, the general processes referred to the recommendations of the American Association of Endodontists for RET [20]. All cases were completed by endodontists experienced in RET.

At the first visit, round burs or fissure burs were used to prepare the access chamber under the isolation of the rubber dam. The pulp chamber and root canal were disinfected by 10-20mL of 1–3% NaOCl with minimal or no mechanical instrumentation. The pulp chamber and root canal were dried with sterile cotton pellet and sterile paper points. Then calcium hydroxide was placed in the root canal. Glass ionomer cement or temporary sealing materials were used to temporarily restore the access cavity.

RET was usually performed 2 weeks later. If the tooth still appeared obvious clinical symptoms, the steps at the first visit were repeated. After removal of temporary restoration, the saline combined with P5 Ultrasound (Satelec, France) was used to rinse the calcium hydroxide by means of the newtron bidirectional oscillation (vibration frequency: 28-36KHZ; intervals of 10 min /5 minutes). The irrigation was finalized with 10–15 mL of 17% EDTA solution, and the tooth was dried with sterile cotton pellet and sterile paper points. The CGF/PRF layer was carefully detached and fragmented. The CGF/PRF fragments were placed in the root canal to the level of cemento-enamel junction (CEJ). The iRoot BP (Innovative Bioceramix, Vancouver, BC, Canada) was placed on the CGF/PRF fragments to form a plug. The access cavity was filled with flowable composite (Beautifil Flow Plus, SHOFO, Japan) and then with packable composite (3M, USA) when the iRoot BP material stiffened after one week.

### Assessment of outcome

The absence of any clinical symptoms with complete resolution of periapical lesion were considered as indicators of healing or success [21]. Given the extended duration required for complete resolution of periapical lesions, clinical success in the study is defined as the absence of any clinical symptoms with the reduction or elimination in the size of the periapical lesion and no need for endodontic retreatment during the recall period.

The root was retrospectively evaluated by pre-operative and follow-up periapical radiographs. Independent analysis was performed in T1 (3–6 months), T2 (7–12 months), and T3 (12–24 months) follow-up [22]. The pre-operative and follow-up periapical radiographs saved in JPG format were opened in the Image J software program (National Institutes of Health, USA) and digitally measured after standardization provided by TurboReg plugin (Swiss Federal Institute of Technology, Switzerland).

**Fig. 5:**
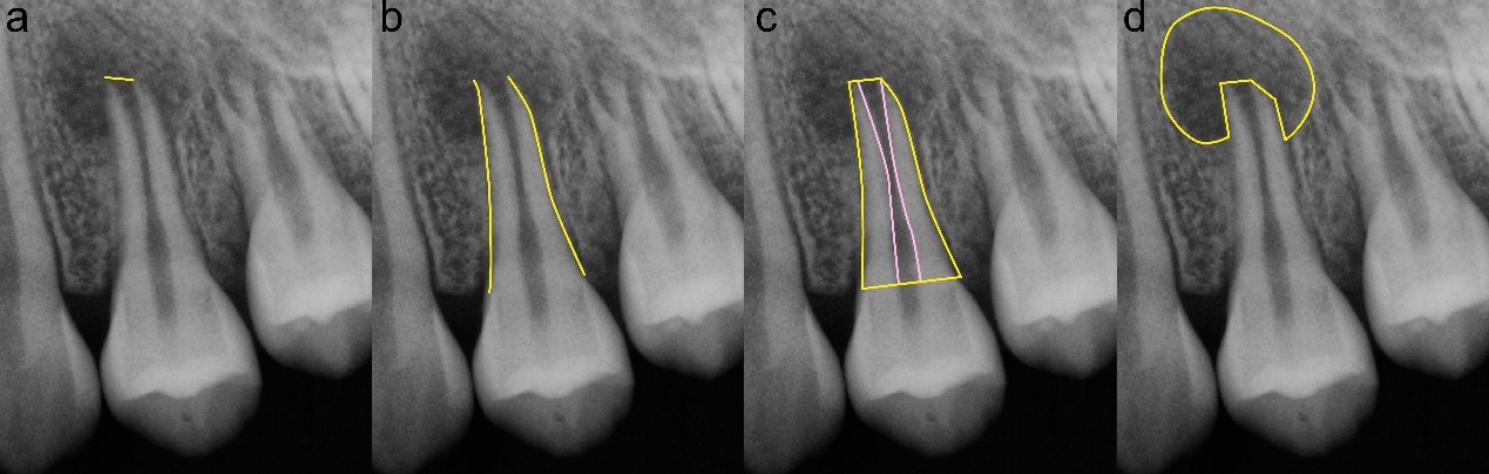
A schematic of digital measurements: **(a)** apical width. **(b)** root length. **(c)** RRA. **(d)** periapical lesion size

The apical foramen diameter, root length, radiographic root area (RRA) and periapical lesion (PAL) were measured referred to the study of Jun et al. [22]. The straight-line tool was used to measure the apical foramen diameter and root length. Apical foramen diameter was measured as the straight line crossing the apex of the root from the mesial to distal. Root length was obtained as the average of 2 straight lines from the mesial and distal cemento-enamel junction to the root apex at the mesial and distal sides. The polygon tool was used to measure RRA and PAL. RRA = root outline under CEJ- root canal space. PAL = the area of periapical area with significantly reduced bone density. The measurement was shown schematically in Fig. 5.

All measurements were performed by two observers using software at a one-week interval and the average of two measurements in each group was recorded. The results of quantitative analysis in periapical area were described in change ratio (%) which was calculated as follows: (preoperative measured value − postoperative measured value) / preoperative measured value × 100%.

## Statistical analysis

SPSS 22.0 software was used to analyze the data. The intraclass correlation (ICC) test was performed to evaluate the reliability of the examiner. When the data coincided with normal distribution, the measured value was expressed as mean ± standard deviation. When the data didn’t coincide with normal distribution, the measured value was expressed as median, first quartile and third quartile. T test and Mann-Whitney test were applied to difference comparison among continuous variables, while dichotomous variables were evaluated by Fisher’s exact test. The significance level was set at P < 0.05.

## Results

The study included 13 teeth from 13 patients (9 Females and 4 males), in which 7 and 6 teeth were treated with CGF and PRF, respectively. The average age of patients in CGF group and PRF group was 11.43 years (10–15 years) and 10.67 years (7–14 years), respectively. The demographic characteristics of the study population were shown in Table 1. There was no significant difference in gender, age, etiology, tooth type, and Nolla stage between the two groups (P > 0.05). The ICCs were over 0.8, which indicated that the measurement made by the observer was reliable over time.

**Table 1:** Demographics and baseline characteristics of the patients

Group	Gender	Age (year)		Aetiology	Tooth position*	Nolla stage
PRF group	Female	14	Trauma		21	9
	Female	7	Developmental dental anomalies		11	7
	Male	8	Developmental dental anomalies		21	7
	Female	9	Developmental dental anomalies		35	7
	Female	15	Developmental dental anomalies		35	9
	Female	11	Developmental dental anomalies		35	9
CGF group	Female	12	Developmental dental anomalies		45	7
	Male	10	Developmental dental anomalies		35	9
	Female	11	Developmental dental anomalies		44	8
	Female	11	Developmental dental anomalies		24	9
	Male	10	No report		35	7
	Male	11	Trauma		21	8
	Female	15	Developmental dental anomalies		45	9

* According to the FDI tooth numbering system

In order to determine whether there were differences between CGF group and PRF group in terms of apical closure, root length, root canal thickness, and periapical lesion, we quantified the changes of preoperative and postoperative periapical radiographs according to follow-up periods. The specific results were shown in Table 2.

**Table 2:** Quantitative radiographic outcomes of the patients [Mean ± standard deviation/median (25-75%)]

Stage	Group	Apical width reduction ratio (%)	Root length increase ratio (%)		RRA increase ratio (%)		Periapical lesion (%)	Follow-up period (month)
T1 (3–6 months)	CGF	10.41(6.33–62.71)	6.06(0.25–10.61)	9.91 ± 6.68		90.30(80.59-)		4.2
	PRF	11.08(4.06–28.41)	2.67(0.57–11.50)	10.06 ± 6.95		100.00(43.21-)		4.17
T2(6-12months)	CGF	62.14 ± 35.83	1.31(0.02-)	5.73(0.99-)		/		7.33
	PRF	24.61 ± 11.68	0.95(0.18-)	25.75(6.36-)		/		8.00
T3(12-24months)	CGF	31.50 ± 6.70	2.33 ± 1.33	12.17 ± 4.66*		/		13.33
	PRF	59.93 ± 39.34	15.74 ± 12.21	45.06 ± 12.30*		/		16.33

T-test was used for values expressed as mean ± SD; Mann-Whitney test was used for values expressed as median, one-quarter value, and three-quarter value. * P < 0.05

In the T1 (3–6 months), all the clinical symptoms of the teeth were eliminated, and for both groups, the success rate of RET were 100%. There was no significant difference between CGF group and PRF group in the reduction rate of apical foramen width, the increase rate of root length, the increase rate of RRA, and the reduction rate of periapical lesions (P > 0.05).

In the T2 (6–12 months), the clinical symptoms of all teeth were eliminated, and for both groups, the success rate of RET were 100%. There was one calcification case in each group, respectively. There was no significant difference between CGF group and PRF group in the reduction rate of apical foramen width, the increase rate of root length, the increase rate of RRA and the reduction rate of periapical lesions (P > 0.05).

In the T3 (12–24 months), the clinical symptoms of all teeth were eliminated, and for both groups, the success rate of RET were 100%. There was no significant difference between CGF group and PRF group in the reduction rate of apical foramen width, the increase rate of root length, and the reduction rate of periapical lesions (P > 0.05). Conversely, the increase rate of RRA of the PRF group was statistically significantly higher than the CGF group (P < 0.05).

## Discussion

In this study, in order to compare the curative effect of each stage objectively, the follow-up time was divided into T1, T2 and T3 stages because of the lack of standardization of case follow-up. The primary goal of RET was to eliminate clinical symptoms and manifestations of periapical lesion healing, which we defined as success. The present study indicated that the success rate of PRF and CGF in each stage of RET was 100%, and there was obvious periapical lesion reduction in the T1 stage. It was consistent with the previous study whose result was that compared with BC, PRF had similar effects on apical closure, dentin wall thickening, apical healing and root lengthening [23, 24]. However, another study revealed that the long-term effect of BC group was stable and the success rate was high, while the long-term success rate of CGF group decreased gradually [25].

Apical closure and root lengthening are important indicators of root development and the thickness of root canal represents the fracture resistance of tooth root. CGF was placed in the root canal of the immature single-rooted teeth in the beagle dogs after pulpectomy and then after 8 weeks the results showed that there was a continuing root development in immature teeth and regenerated pulp-like tissues in root canal [26]. PRF composite dental pulp stem cells (DPSCs) were placed into the root canal and subcutaneously in nude mice. The results showed that the transplantation of DPSC/PRF structure not only promoted the regeneration of pulp-like tissues and the rich distribution of capillaries, but also promoted the deposition of regenerated dentin along the intracanal walls [27]. These in vitro studies showed that both CGF and PRF can promote pulp and dentin regeneration. In this study, the apical foramen closure and the root lengthening of the two groups were facilitated in different degrees, which aligns with findings from certain case reports [28, 29]. There was no significant difference between the two groups, which is consistent with an in vitro study suggesting that the impact on apical papilla stem cells did not show significant differences between CGF and PRF [30].

The results of quantitative measurement of RRA showed that the root canal wall of both CGF group and PRF group had hard tissue growth. Except that the increase rate of RRA in PRF group in the T3 stage was above one in CGF group with statistically significance, there was no significant difference in the increase rate of RRA between the two groups in T1 and T2 stage. This was consistent with the previous finding that PRF more effectively promoted in expression levels of osteogenic/odontoblast-related genes than CGF [30]. Also, the non-standardization of measurements cannot be ruled out as the cause of the discrepancy of RRA in T3 stage. In contrast to this study, Mahendran et al. [31] reported that CGF could significantly promoted root development compared with PRF in early stage, but the benefit diminished at the 18-month follow-up. This difference was attributed to CGF having a denser fibrin matrix and a higher concentration of growth factors than PRF. Additionally, the study by Mahendran et al. utilized cone-beam computed tomography for radiographic assessment, which is more accurate than periapical radiographs. Moreover, the age of the cases included in their study was significantly older than in our study, which could also contribute to the observed differences.

**Fig. 6:**
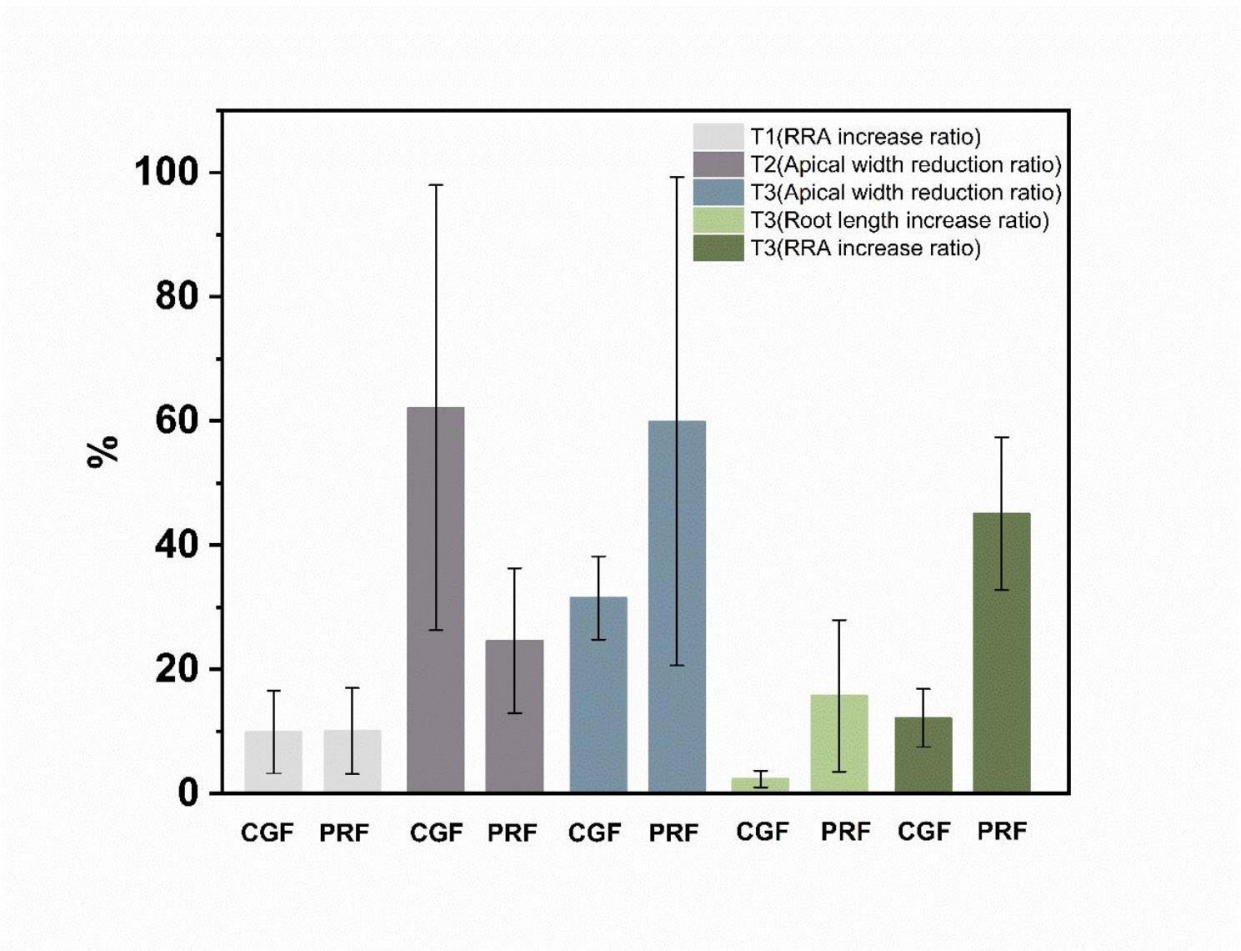
Comparison of apical width reduction ratio, root length increase ratio and RRA increase ratio with mean ± standard deviation in T1, T2 and T3 between two groups

It’s worth noting that the 20% cutoff point of the radiographic increment was usually used as threshold of clinical significance in quantitative analysis of root changes, which was regarded as avoiding overestimation of the negligible root development evidence and the radiographic error. The 20% threshold was not introduced in this study because it was an arbitrary figure and was not based on scientifical testing. Nevertheless, based on this consideration, it was suggested that the present study results should be interpreted conservatively. It is acknowledged that emphasizing quantitative analysis instead of a threshold value may result in a higher standard deviation (Fig. 6), which is consistent with previous studies [29], that also reported a relatively high standard deviation.

Ulusoy et al. conducted a randomized controlled study on the clinical efficacy of PRP, PRF, and BC in dental pulp revascularization and found that the presence of scaffold could significantly reduce the incidence of root canal calcification [32]. In this study, in the T2 stage, there was one case of root canal calcification in CGF group and one case in PRF group. There was no significant difference in improving the complication of calcification between the two groups. Pulp vitality recovery is the third-level goal of RET. It has been observed that pulp vitality recovery is positively correlated with root development [33]. A preliminary study found that the incidence of positive sensitivity response for electric pulp testing in necrotic immature permanent teeth was 25% [34]. Due to the lack of records of sensibility test in medical records, this study lacks the comparison of pulp vitality responses.

In addition to the insufficient number of cases and the inherent bias of retrospective studies, this study also had methodological limitations. One of the limitations is that the process of obtaining platelet derivatives lacks standardization, which could result in errors [35]. And the standardization of measurement depended on ImageJ software and TurboReg plugin. In practice, different angulation of periapical X-ray may lead to vastly different measurement. While this study indicated no significant difference between CGF and PRF regarding the resolution of clinical signs and symptoms, periapical lesion healing, and continued root development as scaffolds in RET, the small sample size was insufficient to draw definitive conclusion. This provided useful information for the selection of scaffolds. Nevertheless, more randomized prospective controlled studies are needed to confirm the ideal scaffold.

## Conclusions

Both CGF and PRF demonstrated similar clinical performance in terms of resolution of clinical signs and symptoms, periapical lesion healing, and continued root development as scaffolds in RET. Further prospective studies with large samples and longer follow-up periods are needed to support the conclusion.

## Data Availability

All data generated or analyzed during this study are included in the article.

## Abbreviations

RET: Regenerative endodontic therapy
CGF: Concentrated growth factor
PRF: Platelet-rich fibrin
RRA: Radiographic root area
PAL: Periapical lesion
CEJ: Cement-enamel junction
DPSCs: Dental pulp stem cells
